# Undetectable Macular Neovascularization on OCT Angiography in Age Related Macular Degeneration: Comparison between Different Devices

**DOI:** 10.3390/medicina58091246

**Published:** 2022-09-08

**Authors:** Meryem Filali Ansary, Emanuele Crincoli, Oudy Semoun, Joel Uzzan, Francesca Amoroso, Camille Jung, Alexandra Miere, Eric Souied

**Affiliations:** 1 Department of Ophthalmology, Creteil University Eye Clinic, Hopital Intercommunal de Creteil, University Paris Est, 40 Avenue de Verdun, 94010 Creteil, France; 2 Center of Clinical Research, Centre Hospitalier Intercommunal de Créteil, 94010 Creteil, France

**Keywords:** optical coherence tomography (OCT), OCT-angiography (OCTA), macular neovascularization, spectral-domain OCTA, swept-source OCTA

## Abstract

*Background and Objectives*: The aim of this study was to report the characteristics of macular neovascularization (MNV) with undetectable flow on optical coherence tomography angiography (OCTA) in neovascular age related macular degeneration (nAMD), and compare them with the characteristics of detectable MNV. *Materials and Methods*: Patients with a diagnosis of nAMD who underwent dye imaging and OCTA in the same day were included and divided into two groups: undetectable and detectable flow on OCTA. Three OCTA devices were used, two with spectral-domain technology (AngioVue, RTVue 100xAvanti, Optovue, Freemont, CA, USA and Heidelberg OCT2 Beta Angiography Module, Heidelberg Engineering, Germany) and one swept-source OCTA (PlexElite 9000; Carl Zeiss Meditec, Inc., Dublin, CA, USA). We studied the demographics, neovascularization characteristics, and OCTA device and acquisition characteristics for both groups. *Results*: A global comparison between Group 1 and Group 2 was made, followed by an analysis of variables associated with (un)detectability for each OCTA device. A total of 108 eyes were included: 90 in the detectable group (Group 1) and 18 in the undetectable group (Group 2), corresponding to a global sensitivity of OCTA for the detection of MNV of 83.49%. There was a statistically significant difference between the two groups regarding MNV type (*p* = 0.02) and PED height (*p* = 0.017). For the three devices, detection sensitivity with automatic segmentation was significantly lower than with manual segmentation. For Heidelberg, PED Height and scan quality explained 68.3% of the undetectability. For AngioVue, PED Height and absence of hemorrhage explained 67.9% of undetectability. *Conclusions*: In this study, we found a global sensitivity of 83.49% for the three OCTA devices combined, with a range from 55.5% to 96.26% depending on the segmentation and OCTA device. This means that undetectable/undetected MNV can represent up to 45% of the examinations, eventually misdiagnosing choroidal neovascularization for 1 out every 2 patients.

## 1. Introduction

Classically, macular neovascularization (MNV) is detected using dye-based angiography, such as fluorescein (FA) and indocyanine green angiography (ICGA), as well as, in recent decades, spectral-domain optical coherence tomography (SD-OCT). We can distinguish three types of MNV in neovascular AMD: type 1 or occult CNV (below the retinal pigment epithelium (RPE), type 2 or classical CNV (above the RPE), and type 3 or retinal-choroidal anastomosis.

While dye-based angiography allows the visualization of the neovascular membrane morphology, accompanied, in the late frames, by leakage (FA) or late plaques (ICGA), SD-OCT enables the visualization of indirect signs of exudation, such as subretinal/intraretinal fluid or subretinal hyper-reflective exudation or material. Dye-based angiography is therefore used to diagnose MNV, while SD-OCT is used for activity detection of the MNV by revealing exudation signs.

Given that different imaging modalities reveal complementary information on the morphology and activity of the MNV, multimodal imaging has become the gold standard for diagnosing and monitoring patients with age-related macular degeneration (AMD) [1,2]. However, FA and ICG angiography are dye-based examinations, and therefore invasive. Furthermore, fluorescein can induce anaphylactic reactions that can be lethal [3,4].

With the advent of OCT angiography (OCTA), however, a non-invasive tool allowing the detailed visualization (and quantification) of MNV emerged. Indeed, in recent literature, OCTA has demonstrated high sensitivity and specificity for the detection of MNVs; Souedan et al. found a sensitivity of 85.62% in 2018 [5,6,7,8].

Moreover, OCTA provides depth-resolved images of blood flow in the retina and choroid. Still, several factors are involved in the visualization process, from image signal strength, artifacts, automatic/manual segmentation to the embedded software, or swept-source/spectral-domain technology of the instrument. Therefore, the detection of MNVs on OCTA depends on the factors mentioned above and the MNV characteristics. In these cases, conventional multimodal imaging is needed to assess neovascularization’s presence correctly.

Therefore, today, the diagnosis of exudative choroidal neovascularization and the decision to treat with anti-vascular endothelial growth factor (VEGF) relies on visual acuity, fundus examination (hemorrhages), SD-OCT, and OCTA [9]. The anti-VEGF treatment is recommended if these explorations lead to a certain, positive diagnosis. However, if the diagnosis is still uncertain, dye-imaging is then required [10].

There are studies on the visibility of MNV in OCTA depending on the height of pigment epithelium detachment (PED), and others on the neovessel type and the difference between spectral-domain and swept-source imaging. This study analyzes the factors involved in the undetectability of MNVs in OCTA and the estimated prevalence.

## 2. Materials and Methods

### 2.1. Patient Selection

In this monocentric study, we retrospectively included patients with neovascular AMD between January 2017 and November 2019 who had undergone an OCTA examination, FA, ICGA, and SD-OCT on the same day in the Department of Ophthalmology of Centre Hospitalier Intercommunal de Creteil. Two groups were distinguished: Group 1, detectable MNV on OCTA, and Group 2, undetectable MNV on OCTA.

This retrospective study was approved by the Institutional Review Board of the Federation France Macula. The research adhered to the tenets of the declaration of Helsinki.

Inclusion criteria were patients with neovascular AMD, confirmed by SD-OCT (Spectralis OCT2 HRA+OCT, Heidelberg Engineering, Heidelberg, Germany), FA, and ICGA. OCTA had to be performed on the same day, using either spectral-domain OCTA (RTVue 100xAvanti, Optovue, Fremon, CA, USA, or Heidelberg Engineering, Germany) or swept-source OCTA (SS-OCTA) (PLEX Elite 9000; Carl Zeiss Meditec, Inc., Dublin, CA, USA).

The presence of MNV on FA was defined as occult leakage or early to late hyperfluorescence. On ICG, the presence of a vascular membrane was distinguished as a late hyperfluorescent plaque in type 1 MNV. On SD-OCT, alterations of the RPE can be observed, with fibrovascular PED in Type 1 MNV and subretinal hyper-reflective material in Type 2 vascular membrane. MNV on OCTA was defined as a high flow network in the outer retinal/avascular layer or the choriocapillaris segmentation, as previously described [11,12].

Exclusion criteria were unclear: media (corneal opacities, advanced cataract), bad quality scan, other causes of choroidal neovascularization, and dye imaging performed more than a week before or after the OCTA examination.

For each patient, we studied the following: demographics, the OCTA device used and its signal strength, the neovessels type, maximal pigment epithelium detachment (PED) height, maximal PED width, hemorrhage, history of previous treatment, maximal retinal thickness, and choroidal thickness. Measures were made using the Heidelberg OCT2 measuring tool system.

### 2.2. Image Acquisition and Analysis

#### OCTA Devices

The OCTA devices used routinely in our department with spectral-domain technology were AngioVue (RTVue 100xAvanti, Optovue, Fremon, CA, USA) and Heidelberg Spectralis OCT2 beta angiography module (Heidelberg Engineering, Heidelberg, Germany). The device with swept-source technology was Plex Elite (9000; Carl Zeiss Meditec, Inc., Dublin, CA, USA).

Optical coherence tomography angiography examinations were performed using a 3 × 3 mm or 6 × 6 mm volume scan pattern to capture the entire lesion. The signal strength needed to be superior to 6/10 on AngioVue and Plex Elite.

The SD-OCTA AngioVue device operates on an 840 nm wavelength and 70,000 A-scans per second to acquire OCTA volumes consisting of 2 repeated B-scans. Since these systems operate at short wavelengths, visualization beneath the retinal pigment epithelium (RPE) may be obscured due to signal attenuation from the RPE-Bruch’s membrane complex [13].

The Heidelberg OCT2 device (Spectralis, Heidelberg Engineering, Heidelberg, Germany) can acquire 85,000 A-scans per second, with an axial resolution of 7 μm, a lateral resolution of 14 μm, and a bandwidth of 50 nm. It operates on an 870 nm wavelength [14].

The SS-OCTA device used is an ultra-high speed long-wavelength prototype that operates at ~1050 nm wavelength and 100,000 A-scans/second. Its longer wavelength and acquisition speed allow an improved immunity to ocular opacity and deeper penetration into the choroid [13].

MNV was first sought with automatic segmentation (outer retina and choriocapillaris on the AngioVue device, avascular layer and choriocapillaris on the Heidelberg OCT2, and outer retinal to choriocapillaris (ORCC), avascular layer or choriocapillaris on the PlexElite 9000), then with a meticulous manual correction of segmentation by experimented physicians (JU and A.M) if necessary. Notably, eyes in both groups underwent one or more OCTA examinations on the same visit and were classified as undetectable (Group 2) if at least one OCTA examination was negative.

### 2.3. Statistics

Statistical analysis was conducted using SPSS software (IBM SPSS Statistics 26.0). Sample size calculation was performed, calculating an 80% power of the study, an alpha error of 5%, and a clinically significant difference in vessel density of 5%. The normality of the distribution for continuous quantitative variables was assessed using the Shapiro–Wilk test. Univariate analysis of normal quantitative variables was performed using a two-tailed *T* test for independent samples. Qualitative variables were compared using a Chi square test with Bonferroni post hoc analysis; a Fisher exact test was used instead when appropriate. Binomial logistic regression was performed to assess odds ratios (ORs) for each considered covariate. The sensitivity of different instruments for MNV detection and sensitivity, with or without manual segmentation of the lesion, were compared using the McNemar–Bowker test. Receiver operating characteristics (ROC) curves were used to define sensitivity, specificity, and cut-off values of quantitative variables in the prediction of the undetectability of the MNV. A *p*-value < 0.05 was considered statistically significant.

## 3. Results

### 3.1. Demographics

A total of 97 patients, mean age 80.9 +/− 8 years were selected according to the inclusion criteria. Of these, 11 patients (11.3%) were treated for bilateral nAMD, leading to a total of 108 eyes. In total, 69% of the patients were women (67/97), and 86.1% were treatment naïve (93/15).

MNVs were undetectable on OCTA in 18/108 eyes, leading to an estimated prevalence of MNV OCTA detection of 83.3%. In Group 1, 90 eyes (83.3%) were included, (detectable MNVs on OCTA) and in Group 2, 18 eyes (16.7%) were included (undetectable MNVs on OCTA). Notably, in Group 2, some patients benefited from more than one OCTA device examination and were considered as false negative if the MNV was undetectable on at least one of them: 9 out of 18 eyes underwent two OCTA examinations on the same day, and 1 out of 18 underwent three OCTA examinations using all three devices (Appendix B).

### 3.2. Global Comparison between Group 1 and Group 2

MNV characteristics were studied: 85% were treatment-naïve in Group 1, while 89% were treatment-naïve in Group 2 (*p* = 0.709). A hemorrhage was present on fundus examination/fundus photography in 51.1% of eyes included in Group 1 versus 21.1% in Group 2 (univariate *p* = 0.022). Regression analysis confirmed the significance of the finding (*p* = 0.023), attesting an OR of 0.164.

Regarding the type of MNV in the two groups, there was a statistically significant difference between the two groups (*p* = 0.02). In Group 1, 41.1% of eyes presented with type 1 MNV (37/90), 30% presented with type 2 MNV (27/90), and 28.9% presented with type 3 MNV (26/90). In Group 2, type 1 MNV was found in 83.3% (15/18), while type 3 MNV represented 16.7% (3/18). There was no type 2 MNV in Group 2.

PED height was 151.9 ± 135 µm (137–189) in Group 1 versus 219.8 ± 128 µm (156–283) (*p* = 0.017) in Group 2. PED height was associated with undetectability with an OR = 1.32. PED width was 1469 ± 1164 µm in Group 1 versus 1485 ± 804 µm in Group 2 (*p* = 0.791). There were no statistically significant differences in central retinal thickness (CRT) and choroidal thickness between the two groups (*p* = 0.453 and *p* = 0.904, respectively). The global comparison between Group 1 and Group 2 is summarized in Table 1.

### 3.3. Variables Associated with MNV (un) Detectability on Different OCTA Devices

A comparison between Groups 1 and 2 about OCTA devices was performed. Notably, on the same visit, some of the eyes included in Groups 1 and 2 underwent ≥ 1 OCTA examination(s) on ≥1 OCTA device. Each OCTA examination (possibly of the same eye) was considered separately for comparing different OCTA devices. Therefore, the 108 eyes included in this study underwent 136 OCTA examinations. In total, 57 OCTA examinations were performed using Heidelberg OCT2 (41.9%), 52 using AngioVue (38.2%), and 27 using PlexElite (19.9%).

#### 3.3.1. Heidelberg OCT2

**Figure 1:**
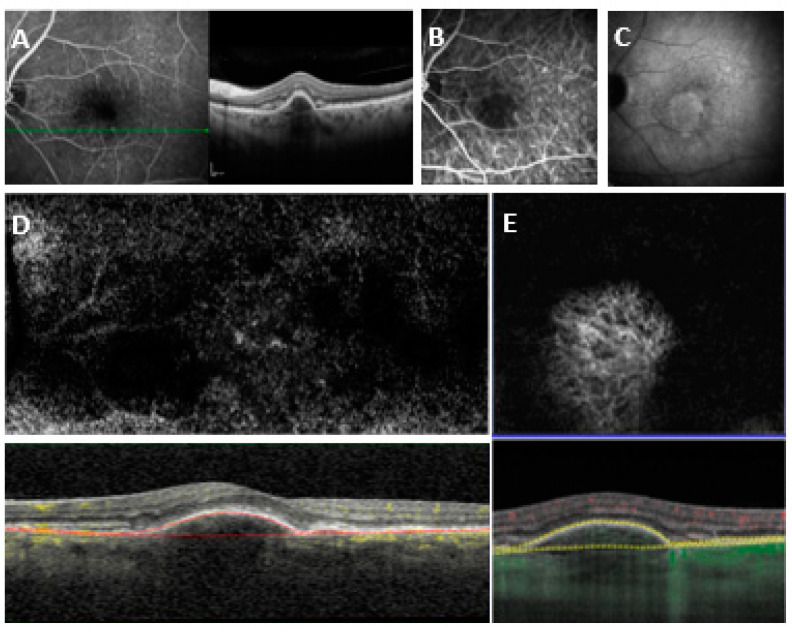
(**A**) SD-OCT crossing through the lesion showing a fibrovascular pigment epithelial detachment (PED) with subretinal fluid. (**B**) Early indocyanine green angiography (ICGA). (**C**) Late ICGA with late hyperfluorescent plaque. (**D**) En face and cross-sectional 3 × 3 mm^2^ SD-OCTA with Heidelberg OCT2 RP fit manual segmentation showing no detectable flow. (**E**) En face and cross-sectional 3 × 3 mm^2^ SS-OCTA with PlexElite RPE-fit manual segmentation showing a distinct high flow network.

For Heidelberg OCT2, detection sensitivity with automatic segmentation (avascular layer and choriocapillaris) was 68.42% (39/57) (AUROC = 0.724, specificity = 84.2%, PPV = 78.0%, NPV = 72.7%), and sensitivity with manual segmentation was 87.71% (50/57) (AUROC = 0.834, specificity = 91.2%, PPV = 90.9%, NPV = 88.1%) (*p* = 0.037). A binomial logistic regression model for quantitative and qualitative variables was performed to detect variables associated with undetectability. PED height was significantly higher in Group 2 (289.67 ± 70.9, *n* = 7 examinations) compared to Group 1 (212.70 ± 139.7, *n* = 50 examinations) and was associated in a statistically significant manner with undetectability (*p* = 0.017, odds ratio 1.52). PED height proved to be a statistically significant parameter for predicting MNV undetectability with OCTA (AUROC = 0.7683 (CI 0.625–0.912), *p* = 0.0354). In particular, PED height > 234 µm has a sensitivity of 83.33%, a specificity of 65.85%, and an LR = 2.440 in the prediction of MNV undetectability, while a PED height > 283 µm has an 80.49% specificity in predicting MNV undetectability (see Figure 1).

Moreover, scan quality was significantly higher in Group 1 (mean 34.36 +/− 4.20 dB, n = 50 examinations) compared to Group 2 (mean 30.43 +/− 2.38 dB, n = 50 examinations) and was associated in a statistically significant manner with undetectability (*p* = 0.011, odds ratio 2.49). In contrast, hemorrhage was not significantly associated with undetectability according to regression analysis (*p* = 0.279). The binomial logistic regression model was statistically significant (*p* < 0.001) and explained 68.3% of the variance (Nagelkerke R2). 

#### 3.3.2. Optovue OCTA

**Figure 2:**
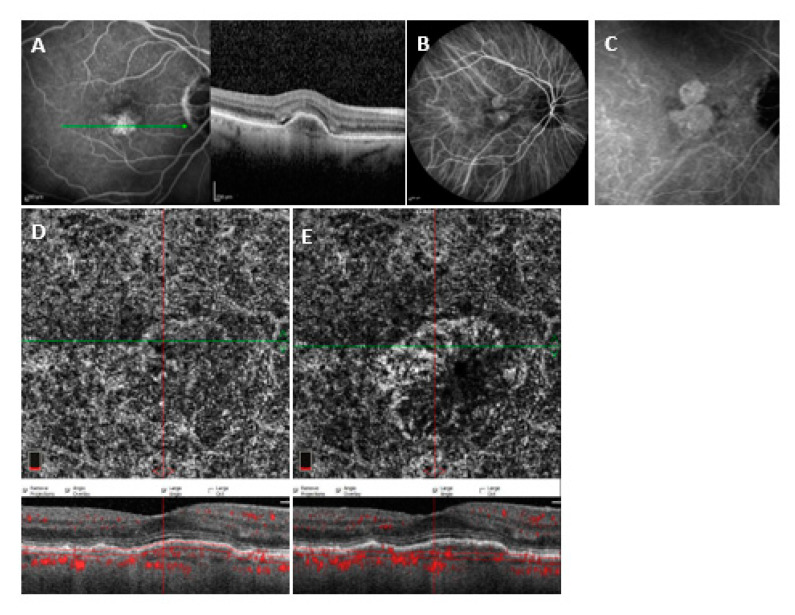
(**A**) SD-OCT crossing through the lesion showing a fibrovascular PED. (**B**) Early indocyanine green angiography. (**C**) Late indocyanine green angiography with late hyperfluorescent plaque. (**D**) En face and cross-sectional 3 × 3 mm^2^ SD-OCTA with ANgiovue automated segmentation showing no distinctable flow. (**E**) En face and cross-sectional 3 × 3 mm^2^ SD-OCTA with Angiovue corrected manual segmentation showing a distinct neovascular complex.

**Figure 3:**
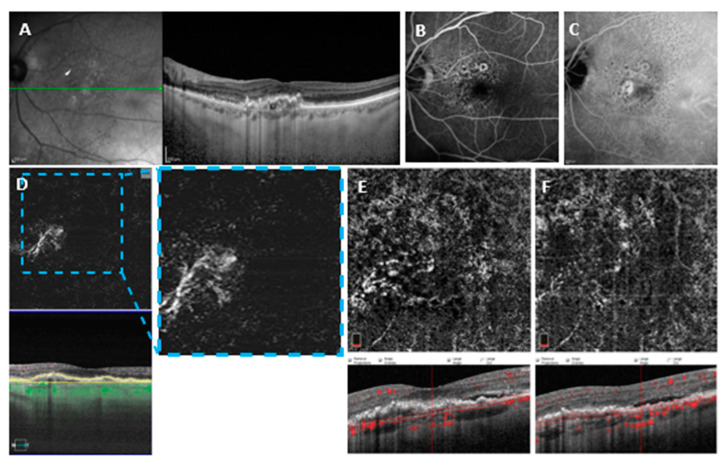
(**A**) SD-OCT crossing through the lesion showing a flattened irregular PED. (**B**) Early fluorescein angiography. (**C**) Late indocyanine green angiography with late hyperfluorescent plaque. (**D**) En face and cross-sectional 6 × 6 mm^2^ SS-OCTA with PleXelite MS Custom segmentation showing distinct flow overlay. (**E**) En face and cross-sectional 3 × 3 mm^2^ SD-OCTA with AngioVue corrected manual segmentation. (**F**) En face and cross-sectional 3 × 3 mm^2^ SD-OCTA with AngioVue automated segmentation showing no detectable flow.

For AngioVue, sensitivity with automatic segmentation (outer retina and choriocapillaris) was 59.6% (31/52) (AUROC = 0.658, specificity = 76.9%, PPV = 72.1%, NPV = 65.6%) and sensitivity with manual segmentation was 76.9% (40/52) (AUROC = 0.791, specificity = 86.5%, PPV = 85.1%, NPV = 78.9%) (*p* = 0.023). A binomial logistic regression model for quantitative and qualitative variables was performed to detect variables associated with undetectability. PED height was significantly higher in Group 2 (223.27 ± 105.30.9, *n* = 12) compared to Group 1 (169.59 ± 84.11, *n* = 40) and was associated in a statistically significant manner with undetectability (*p* = 0.038, odds ratio 1.97). Nevertheless, PED height did not show a significant sensitivity and specificity in the prediction of MNV undetectability (AUROC = 0.6734 (CI 0.4945–0.8523), *p* = 0.0974). Moreover, the presence of hemorrhage was significantly higher in Group 1 (24/40) compared to Group 2 (1/12) and was associated in a statistically significant manner with detectability (*p* = 0.018, odds ratio 0.016). The binomial logistic regression model was statistically significant (*p* < 0.001) and explained 67.9% of the variance (Nagelkerke R2). The type of neovascularization was also significant in MNV visualization using the AngioVue device; type 1 neovascularization represented 83.3% of MNVs in Group 2 versus 40.2% in Group 1 (*p* = 0.01) (see Figure 2 and Figure 3).

#### 3.3.3. PlexElite OCTA

**Figure 4:**
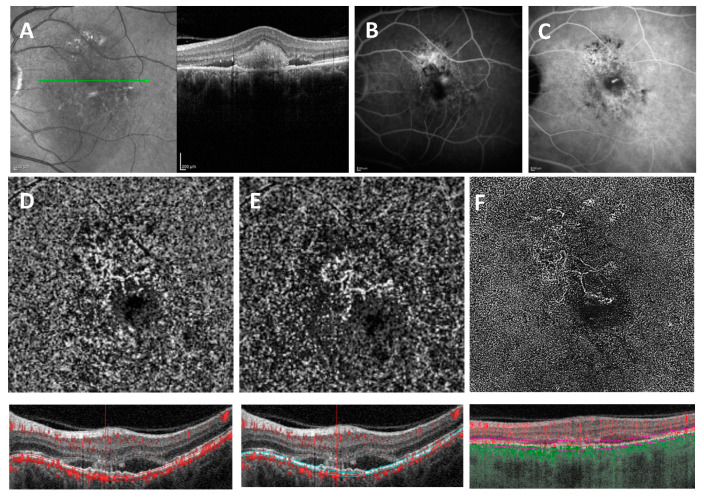
(**A**) SD-OCT crossing through the lesion showing a flattened irregular PED with sub-retinal hyper-reflective material. (**B**) Early fluorescein angiography. (**C**) Late indocyanine green angiography with late hyperfluorescent plaque. (**D**) En face and cross-sectional 6 × 6 mm^2^ SD-OCTA with AngioVue automated segmentation showing no distinct detectable flow. (**E**) En face and cross-sectional 6 × 6 mm^2^ SD-OCTA with AngioVue corrected manual segmentation. (**F**) En face and cross-sectional 6 × 6 mm^2^ SS-OCTA with PlexElite MS Custom segmentation showing distinct flow overlay.

**Figure 5:**
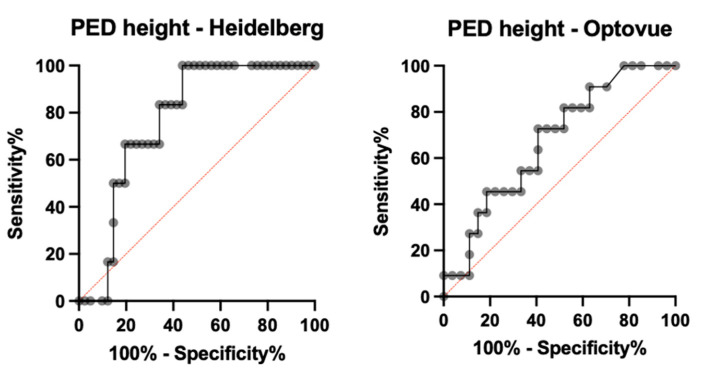
ROC curves for sensitivity and specificity of PED height in the prediction of MNV unpredictability with OCTA when measured with Heidelberg (**left side**) and Angiovue (**right side**) OCT B scan. OCT = optical coherence tomography; PED = pigment epithelial detachment; ROC = receiver operating characteristics curves.

Out of 27 PlexElite examinations, on 1 examination the MNV was undetectable. The sensitivity of detections with automatic segmentation (ORCC, avascular layer and choriocapillaris) was 55.5% (15/27) (AUROC = 0.668, specificity = 74.1%, PPV = 68.2%, NPV = 62.5%), while the sensitivity with manual segmentation was 96.26% (26/27) (AUROC = 0.969, specificity = 96.3%, PPV = 96.3%, NPV = 96.3%) (*p* < 0.001) (see Figure 4 and Figure 5).

## 4. Discussion

In this study, we found an overall sensitivity of 96.3% for the PlexElite device, 87.9% for the Heidelberg OCT2, and 76.9% for AngioVue detection of MNV. The superiority of SS technology for detecting MNVs has been demonstrated by multiple studies [13,15,16], particularly for Type 1 MNV. RPE contains melanin whose absorption and scattering decreases with increasing wavelength [17]. Swept-source technology provides a longer wavelength (1050 nm) and acquisition speed, allowing improved visualization of structures located under the RPE and into the choroid. Nevertheless, using automatic segmentation, the sensitivity of PlexElite was 70.3%. In contrast, after manual segmentation by an expert reader, the sensitivity increased to 96.3%, which is the highest reported in the literature to the best of our knowledge. This may be because, in our study, we included all types of MNVs. Moreover, in our study, only 19.9% of examinations were performed using SS-OCTA. Recent literature found, for the same SS device, a sensitivity of 68% [15] in 2016, 75.7% in 2017, and 80.7% [18] in 2019. These results imply that a learning curve exists and that visualization of MNVs is highly dependent on the clinician’s level of expertise.

The higher sensitivity of the Heidelberg OCT2 compared to the AngioVue was demonstrated in a study [19], explained by both a slightly higher wavelength (870 and 840 for the Heidelberg OCT2 and the AngioVue, respectively) and a higher number of A-scans per second (85,000 and 70,000 for Heidelberg OCT2 and AngioVue, respectively) (Appendix A) [16].

With regard to the sensitivity of each device depending on MNV type, type 1 MNV visualization achieved a sensitivity of 100% (*p* = 0.444) using the SS-OCTA device (PlexElite), 78.6% using the Heidelberg OCT2 (*p* = 0.09) and 61.6% using the AngioVue (*p* = 0.03). Notably, in our study, the detection of type 1 MNVs using the AngioVue was slightly lower than the current literature [6,20,21,22], with the overall detection rates varying from 32% to 90% [13,23]. 

Regarding type 2 MNV, 100% of this type of MNVs were detected by all OCTA devices, independently of the SD- or SS-technology. This fact is easily explained by the subretinal location of these MNVs, allowing a clear visualization using all OCTA systems.

Arrigo et al. found similar results; after calculating the mean reflectivity of both types of MNV, type 2 lesions were detected significantly more often on OCTA than type 1 MNV, with a more evenly distributed flow signal [24].

This study analyzed factors associated with MNV undetectability, leading to false-negative OCTA examinations. For the Heidelberg OCT2, a false negative OCTA is indeed associated with Scan Quality (*p* = 0.02), and PED Height (*p* = 0.017), while the presence of hemorrhage did not reach statistical significance. Moreover, PED height proved to provide a significant cut-off for prediction of MNV undetectability (AUROC = 0.7683 (CI 0.625–0.912), *p* = 0.0354). In particular, a PED higher than 283 μm confers an 80.5% probability of MNV being undetected by OCTA when images are acquired with the Heidelberg OCT2. With the AngioVue, false negative OCTA depended on MNV type, as discussed above, but also on the PED Height (*p* = 0.02) and, surprisingly, the absence of hemorrhage (*p* = 0.03). The scan quality was not significantly associated with MNV visualization, unlike the results of the COFT-1 study [20]. However, in our study, inclusion criteria involved a minimum signal strength of 6/10 for the AngioVue (mean in this study of 0.67 versus 0.59 in COFT-1), which explains why scan quality does not seem to be associated with visualization on this specific device.

The association of the undetectability of MNVs with PED height on OCTA has been noted in the literature [13,20,21]. The presumed rationale is the enhanced masking properties of elevated RPEs, which results in a particular attenuation of OCT signal and, therefore, precludes the visualization of (neo)vascular structures beyond the RPE layer [25,26,27]. Mrejen et al. compared a group of patients presenting with a PED height > 250 µm with a second group presenting a PED height < 250 µm: detectability using the SD-OCTA device was 56.1% and 100%, respectively [27].

While the presence of hemorrhage is usually associated with a lack of MNV visualization [5,9], in our study, there was a higher prevalence of hemorrhages in the detectable group (Group 1) versus the undetectable group (Group 2) for the AngioVue, suggesting that the presence of a retinal hemorrhage alone does not preclude visualization of the MNV in all cases. Moreover, for the Heidelberg OCT2 there was no statistically significant difference in MNV detection associated with the presence of hemorrhage. Consistent with our findings, previous studies such as Jia et al. [28] excluded eyes with subretinal hemorrhage > 50% of the neovessels lesion and found that small hemorrhages did not impact the visibility of the neovessels on the SD-OCTA. A possible explanation for our finding is that the presence of hemorrhages is likely to be associated with larger vessels within MNV, more likely to be detected by OCTA.

Interestingly, 86.2% of the total eyes included in this study were treatment naïve (89% of Group 1 and 85% of Group 2, respectively). The department’s protocol can explain this regarding the first visit for patients with neovascular AMD, which includes SD-OCT, FA, ICG Angiography, and OCTA. While the visualization of MNVs may be dependent on treatment-naïve/treated status, in our study, this variable was not associated with MNV visualization or lack thereof. Previous studies, such as COFT-1, suggested that treated MNVs would gradually develop a fibrovascular complex, making them more visible on OCTA [6,20].

The limitations of this study include its retrospective nature, which explains the unequal distribution between the use of the three OCTA devices and leads to a lack of imaging with all three OCTA devices on the same visit. Moreover, the detection of MNV with OCTA is dependent on the reader, who needs to be trained to implement manual segmentation when required. Last but not least, the binary classification of MNVs into “detectable” (Group 1) and “undetectable” (Group 2) may be a limitation in itself, as some of the included eyes had very distinct MNVs, while others were less evident.

The principal strength of this study is the presence of a control group of detectable MNVs, providing a clear comparison and reliable results concerning the variables associated with MNV (un)detection.

## 5. Conclusions

In this study, we found a global sensitivity of 83.49% for the combined OCTA devices, ranging from 55.5% to 96.26%, depending on the segmentation and the OCTA device. This means undetectable/undetected MNV can represent up to 45% of the examinations, misdiagnosing choroidal neovascularization for one out of every two patients.

The long-term purpose of this study would be to reach an algorithm for decision-making on whether the use of dye imaging is necessary, according to the different parameters (MNV and OCTA device characteristics).

## Figures and Tables

**Table 1:** Comparison of the demographic characteristics, SD-OCT and OCTA findings in both false negative and control groups.

	Group 1	Group 2	*p*	Total
**Total Eyes *n*, (%)**	90 (83.3%)	18 (16.7%)	N/A	108
**Age, years, mean ± SD**	80.7 ± 8.2	82.1 ± 7	0.450	80.9 ± 8
**Sex (Female/Male)** ***n*, (%)**	60/30 67%	15/3 83%	N/A	75/33 69.7%
**Eye (Right/Left)** ***n*, (%)**	39/51 (43%/57%)	7/11 (39%/61%)	0.726	46/62 (42.6%/57.4%)
**Naïve/Not** ***n*, (%)**	77/13 (85%/15%)	16/2 (89%/11%)	0.709	93/15 (86.1%/13.9%)
**Hemorrhage** ***n*, (%)**	48 (53%)	4 (22%)	0.016 *p* Val < 0.05	52 (48.2%)
**Neovessels *n*, (%)**				
**Type I**	37 (41.1%)	15 (83.3%)	0.02	52 (48.1%)
**Type II**	27 (30%)	0	*p* Val < 0.05	27 (25%)
**Type III**	26 (28.9%)	3 (16.7%)		29 (26.9%)
**PED Height, µm, mean ± SD** **95% Conf. Interval, µm**	151.9 ± 135 (137–189)	219.8 ± 128 (156–283)	0.0163 *p* Val < 0.05	163.23 ± 135.52
**PED Width, µm, mean ± SD**	1469 ± 1164	1485 ± 804	0.791	1471 ± 1109
**MRT, µm, mean ± SD**	451 ± 129	474 ± 107	0.453	455 ± 125
**Choroidal Thickness**	187 ± 90	177 ± 71	0.904	185 ± 87
**OCTA Device *n*, (%)** **Heidelberg OCT2** **AngioVue** **PlexElite**	51 (43.6%) 40 (34.2%) 26 (22.2%)	7 (35%) 12 (60%) 1 (5%)		58 (42.3%) 52 (37.9%) 27 (19.8%)

## Data Availability

The datasets of the current study are available from the corresponding author upon reasonable request.

## Appendix A. Technical Characteristics of the Three Different OCTA Systems

System	AngioVue™	Spectralis^®^ OCTA	SS OCT Angio™
Manufactory	OptoVue, Fremont, CA, USA	Heidelberg Engineering, Heidelberg, Germany	9000; Carl Zeiss Meditec, Inc., Dublin, CA, USA
Algorithm	SSADA	Full-spectrum probabilistic approach	OCTARA
OCT device	RTVue XR AVANTI Widefield; SD-OCT	Spectralis OCT2; SD-OCT	PlexElite
Optical source (nm)	Centered on 840 with a bandwidth of 50	Centered on 870 with a bandwidth of 50	Tunable laser centered on 1050 (invisible)
Scan speed (A-scan/s)	70,000	85,000	100,000
